# DIGITAL SOCIAL PRESCRIBING LITE: ADDRESSING BARRIERS OF PREVENTIVE INTEGRATED CARE THROUGH COMMUNITY ENGAGEMENT

**DOI:** 10.1093/geroni/igae098.4163

**Published:** 2024-12-31

**Authors:** Daniel Gan, Adam Hoverman, Kate Mulligan

**Affiliations:** University of Toronto, Toronto, Ontario, Canada; University of Washington, University of Washington, Washington, United States; University of Toronto, University of Toronto, Ontario, Canada

## Abstract

Digital social prescribing solutions attempt to integrate health and social care by targeting both preventive and complex health challenges. However, a lack of community engagement impedes referral uptake. This paper uses “customer discovery” to explore and address current barriers of digital social prescribing from the perspectives of service users and staff of health and community sectors. Phase 1 prioritized community perspectives and led to a community-generated place-based wellness model to align interests across sectors. Phase 2 conducted market research with over 70 health and community stakeholders in Metro Vancouver through snowball sampling. Insights were gathered on current referral processes, barriers to social prescribing, and stakeholder perspectives. From the insights shared, it was evident that achieving integrated care faces twin challenges of resource allocation and staff burnout. Funding-related barriers include inadequate partnership, intermittent funds, lack of billable codes, variations in cost, and lack of monitoring and evaluation. Staffing-related barriers include time-intensive processes, outdated information, inappropriate referrals and lack of guidance for quality improvement. Based on insights from stakeholders, initial cross-sectoral implementation may fruitfully focus on preventing the effects of social isolation. This “lite” version of digital social prescribing requires an automated referral and monitoring platform which allows prevention for the masses while flagging complex cases for intervention. This reduces clinician burden and empowers community sector to support wellbeing. The place-based model “Connectedness, feeling At home, and joyful Play” was incorporated into a web app. Digital social prescribing lite may pave the way for data-driven, organic scaling up of preventive integrated care.

